# Draft genome sequence data of a 4-nitrophenol- degrading bacterium, *Pseudomonas alloputida* strain PNP

**DOI:** 10.1016/j.dib.2021.107390

**Published:** 2021-09-20

**Authors:** Pankaj Kumar Arora, Raj Shekhar Saroj, Rupali Mishra, Rishabh Anand Omar, Puja Kumari, Alok Srivastava, Sanjay Kumar Garg, Vijay Pal Singh

**Affiliations:** a Department of Environmnetal Microbiology, Babasaheb Bhimrao Ambedkar University, Lucknow 226025, India; b Department of Plant Science, Faculty of Applied Sciences, MJP Rohilkhand University, Bareilly, India

**Keywords:** 4-Nitrophenol, Average nucleotide identity, Genome, Sequencing, Bacteria, *Pseudomonas*

## Abstract

A 4-nitrophenol-degrading bacterial strain PNP was isolated from pesticide-contaminated soil collected from Lucknow. Strain PNP utilized 0.5 mM 4-nitrophenol as its carbon source and degraded it completely within 24 h with stoichiometric release of nitrite ions. Strain PNP was associated with the genus *Pseudomonas* in a phylogentic tree and exhibited highest 16S rRNA gene sequence similarity to *Pseudomonas juntendi* BML3 (99.79%) and *Pseudomonas inefficax* JV551A3 (99.79%). Based on values of average nucleotide identity and digital DNA-DNA hybridization among strain PNP and its closely related type strains, it concluded that strain PNP belongs to *Pseudomonas alloputida.* The Illumina HiSeq platform was used to sequence the PNP genome. The draft genome sequence of *Pseudomonas alloputida* PNP was presented here. The total size of the draft assembly was 6,087,340 bp, distributed into 87 contigs with N50 value of 139502. The genome has an average GC content of 61.7% and contains 5461 coding sequences and 77 putative RNA genes. This Whole Genome Shotgun project has been submitted at DDBJ/ENA/GenBank under the accession JAGKJH000000000.

## Specifications Table

**Table:** 

Subject	Microbiology
Specific subject area	Environmnetal Microbiology
Type of data	Data were presented in FASTA format, figures, and tables
How data were acquired	Illumina HiSeq system was used to generate genome sequence data
Data format	Raw, analysed and assembled genome sequences
Parameters for data collection	A pure culture of *Pseudomonas alloputida* PNP was obtained and cultivated and its DNA was isolated and sequenced.
Description of data collection	Genome sequencing, assembly, and annotation. Genome sequencing was performed using HiSeq platform and the Unicycler v0.4.8 was used for initial assembly. Annotation was performed using the NCBI Prokaryotic Genome Automatic Annotation Pipeline and the RAST server.
Data source location	*Pseudomonas alloputida* PNP was isolated from soil sample collected from a pesticide contaminated area, Lucknow (26°51′N 80°57′E ), India
Data accessibility	Data is publicly available at the NCBI Genbank from the following links: https://www.ncbi.nlm.nih.gov/nuccore/JAGKJH000000000 https://www.ncbi.nlm.nih.gov/bioproject/PRJNA717186 https://www.ncbi.nlm.nih.gov/biosample/SAMN18489966

## Value of the Data

• The *Pseudomonas alloputida* PNP genome sequence could reveal important details on degradation of 4-nitrophenol and other xenobiotics.
• The data could be useful for reserchers working on biodegradation and bioremediation of various aromatic compounds.
• This genome information could be useful for comparative genomic research of *Pseudomonas* strains with biodegradation capability.

## 1 Data Description

**Fig. 1:**
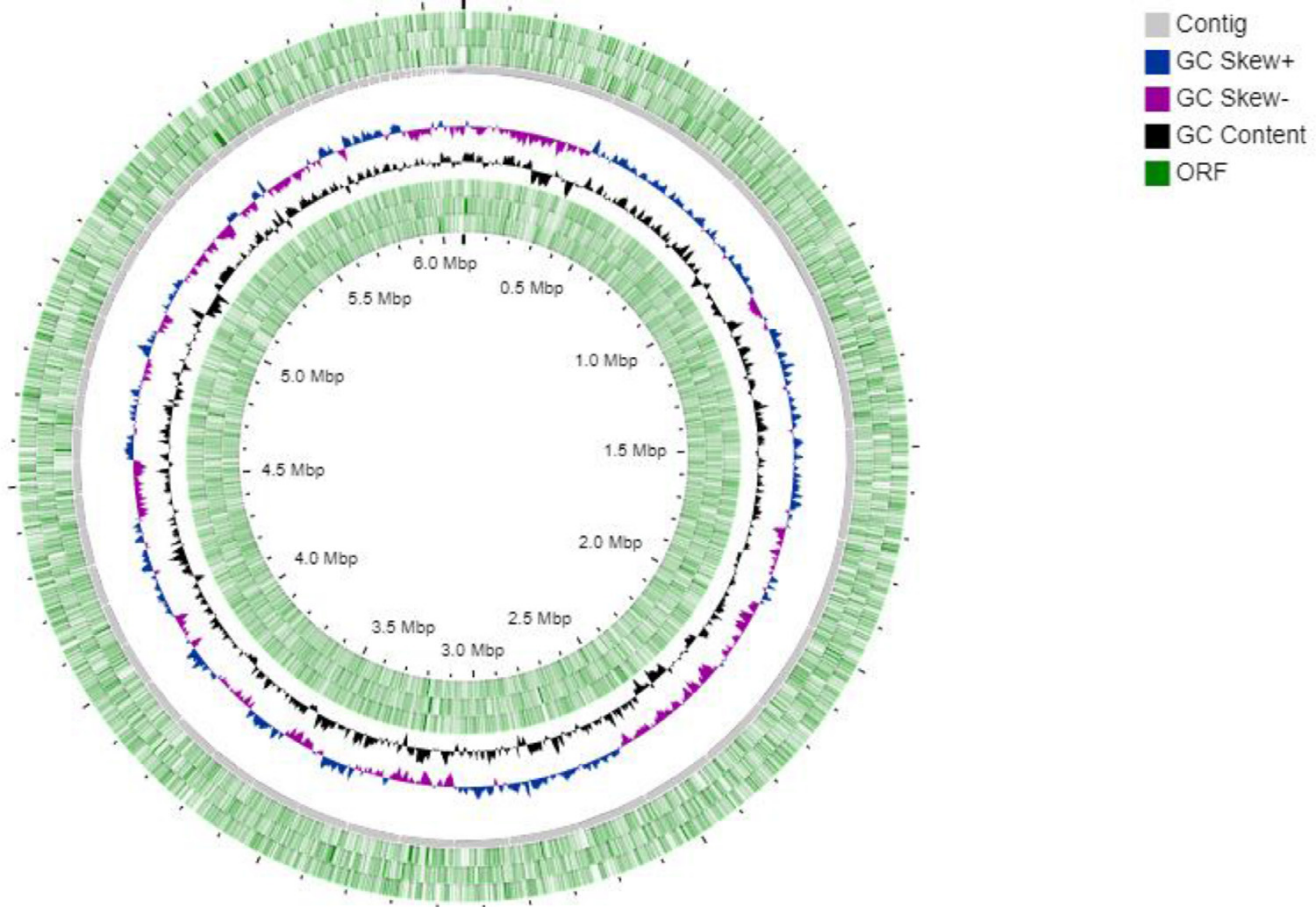
*Pseudomonas alloputida* PNP circular map with ORFs (green colour), Contigs (sleti colour), Positive GC Skew (blue clour), Negative GC Skew (violet colour), and GC content (Black Colour). (For interpretation of the references to color in this figure legend, the reader is referred to the web version of this article.)

*Pseudomonas alloputida* PNP was isolated from the pesticide-contaminated soil collected from Lucknow, India. Strain PNP utilized 4-nitrophenol as its carbon source, totally degrading it in 24 hours and releasing stoichiometric levels of nitrite ions. Table 1 summarizes genomic characteristics of *Pseudomonas alloputida* PNP. The assembled genome of *Pseudomonas alloputida* PNP contained 87 contigs with a total length of 6,087,340 bp and N50 value of 139,502. The G+C content of genome was 61.7%. The NCBI Prokaryotic Genome Automatic Annotation Pipeline (PGAAP) predicted a total of 5635 genes, 5461 of which were associated with coding specific proteins while 77 and 97 of which were responsible for coding RNA genes (69 tRNAs, 5 ncRNAs, 3 16S-23S-5S rRNAs) and pseudogenes, respectively. Fig. 1 shows a circular map of the *Pseudomonas alloputida* PNP genome.

**Table 1:** Genome charcteristcs of *Pseudomonas alloputida* PNP.

Features	Value	Percentage
Number of contigs	87	100
Genome size	6,087,340 bp	100
G+C	3753064 bp	61.7
Genes (total)	5635	100
Protein coding genes	5461	96.91
RNA genes	77	1.36
5S rRNA gene	1	0.02
16S rRNA gene	1	0.02
23S rRNA gene	1	0.02
tRNAs	69	1.22
ncRNAs	5	0.08
Pseudo Genes (total)	97	1.72

**Fig. 2:**
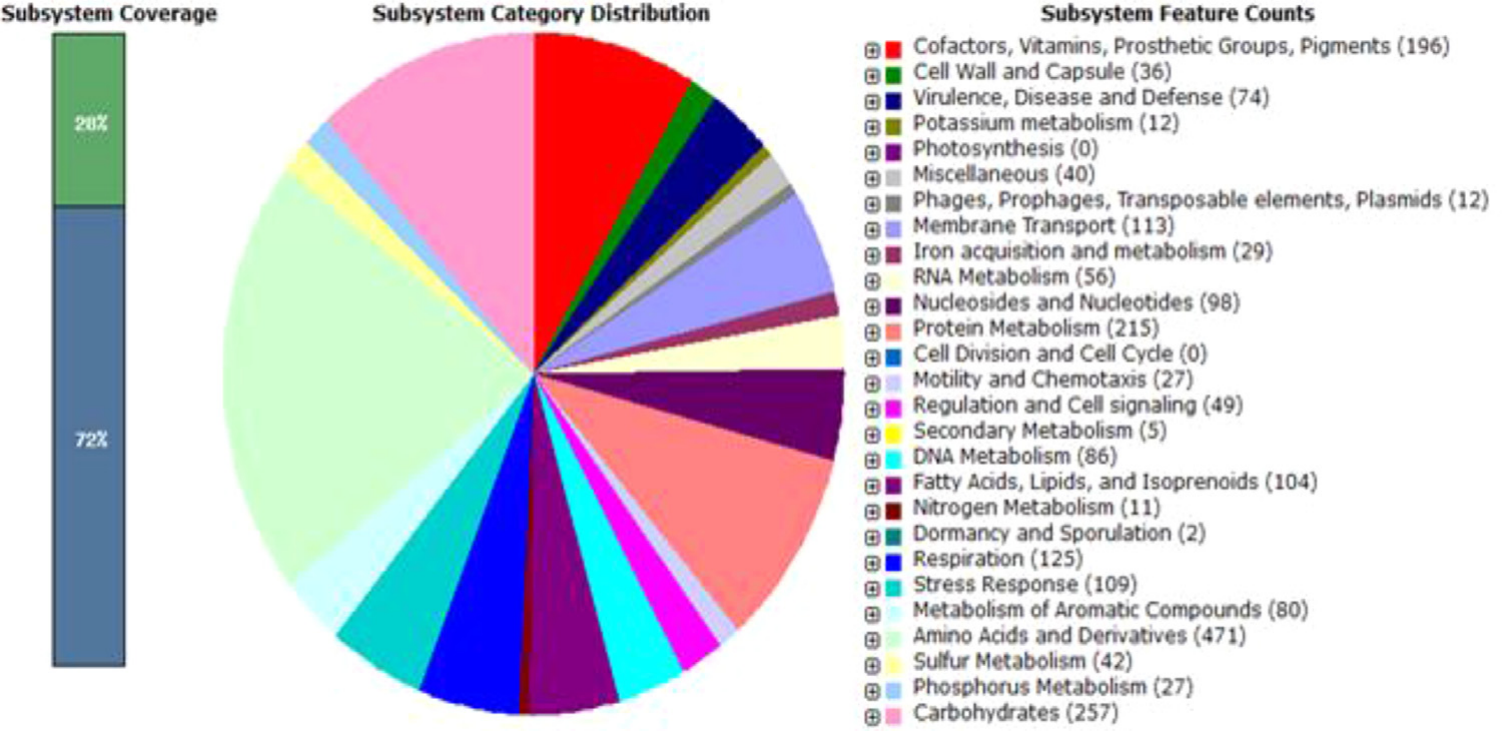
RAST server- based annotation of genome of *Pseudomonas alloputida* PNP.

The annotation of the *Pseudomonas alloputida* PNP genome using RAST server predicted a total of 5726 coding sequences which were categorized into 371 subsystems with 28% subsystem coverage (Fig. 2). Subsystem category belonging to amino acids and derivatives contained highest number of genes (471) followed by carbohydrates (257 ), protein metabolism (215) cofactors, vitamins, prosthetic group, and pigments (196 ), respiration (125), membrane transport (113), stress response (109) and fatty acids, lipids, and isoprenoids (104). Subsystem category “metabolism of aromatic compounds” conatnined 80 genes associated with degradation of benzoate, 4-hydroxybenzoate, quinate, n-phenylalkanoic acid, gentisate, homogentisate, catechol, and protocatechuate. Furthermore, we have also identified genes responsible for hydroxyquinol 1,2-dioxygenase and maleylacetate reductase, which are involved in the lower route of degradation pathway of 4-nitrophenol in Gram-negative bacteria [1]. Furthermore genes involved in bioremediation of chromium and arsenic such chromate efflux transporter, AraC family transcriptional regulator, arsenate reductase ArsC, metalloregulator ArsR/SmtB family transcription factor, arsenical resistance protein ArsH, organoarsenical effux MFS transporter ArsJ, arsenical efflux pump membrane protein ArsB were also detected. Based on annotated data, strain PNP may be used for study of biodegradation of various aromatic compounds as well as biotransformation of chromium and arsenic.

**Fig. 3:**
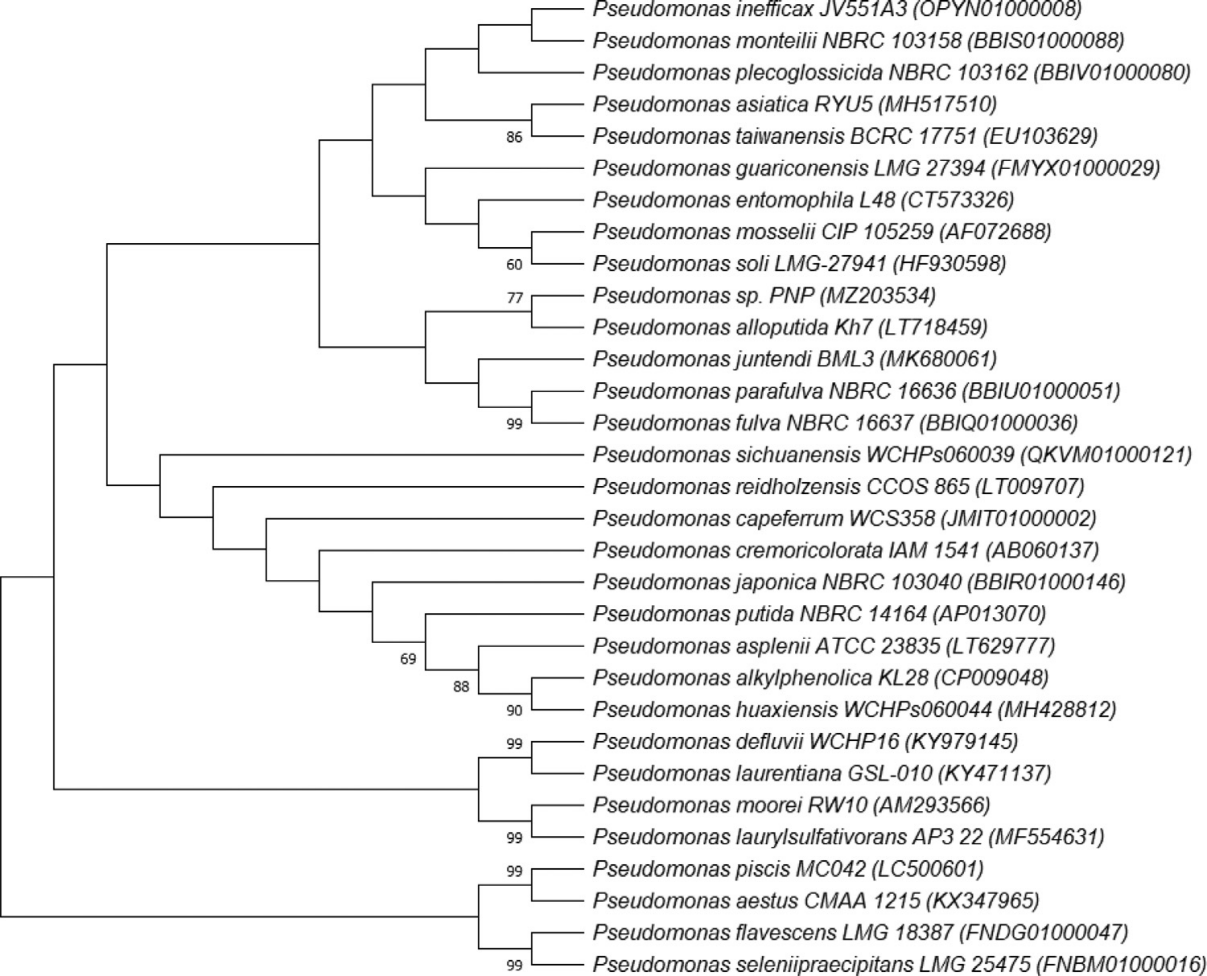
Phylogenetic tree based on 16S rRNA gene sequences of *Pseudomonas alloputida* PNP and its closlely related type strains.

The 16S rRNA gene sequence of strain PNP has been submitted to the NCBI Genbank database under accession number MZ203534. Strain PNP had the highest 16S RNA gene sequence similarity to *Pseudomonas juntendi* BML3 (99.79 %) and *Pseudomonas inefficax* JV551A3 (99.79 % ), followed by *Pseudomonas monteilii* NBRC 103158 (99.73%), *Pseudomonas plecoglossicida* NBRC 103162 (99.73%), *Pseudomonas asiatica* RYU5 (99.73%), *Pseudomonas taiwanensis* DSM 21245 (99.72%), *Pseudomonas entomophila* L48 (99.66%) and *Pseudomonas alloputida* Kh7 (99.45%). Phylogenetic analysis based on 16S rRNA gene sequences of strain PNP and its closely relative strains showed that strain PNP fell within same clade with *Pseudomonas alloputida* Kh7 (Fig. 3). Additinally, whole-genome comparisons, using average nucleotide identity and digital DNA-DNA hybridization tests, indicated that strain PNP belongs to *Pseudomonas alloputida*. Table 2 shows that average nucleotide identity and digital DNA-DNA values amongs strain PNP and closest reference type strains. The average nucleotide identity and digital DNA-DNA values between strain PNP and *Pseudomonas alloputida* Kh7 were 97.34% and 77.90% respectvely. These values were higher than the suggested threshold values for the species delineation (95–96% for ANI and 70% for DDH). Therefore, strain PNP was a new strain of *Pseudomonas alloputida.*

## 2 Experimental Design, Materials and Methods

### 2.1 Sample collection and bacterial isolation

A 4-nitrophenol-mineralizing bacterial strain PNP was isolated from the pesticide contaminated soil collected from Lucknow, India by an enrichment method. For enrichment, 1 g of the collected soil sample was suspended into 1000 ml Erlenmeyer flask containing 250 ml minimal media and 0.5 mM 4-nitrophenol as the sole source of carbon and energy. The media colour was yellow due to the presence of 4-nitrophenol. The flask was incubated at at 30 °C till decolourization of yellow colour of 4-nitrophenol. After decolourization, culture media was serially diluted and plated on minimal media agar plates containing 0.5 mM 4-nitrophenol. The plates were then incubated at 30 °C for 72 h. One bacterial strain designated PNP was selected due to its potential to degrade and decolourize 4-nitrophenol. This strain was preserved in 10 % glycerol vial at -80 °C and used for this study.

### 2.2 Bacterial growth, 4-nitrophenol degradation and nitrite release

Strain PNP was grown on 500 ml Erlenmeyer flask containing 100 ml minimal media contanining 0.5 mM 4-nitrophenol as its sole source of carbon and energy. The flask was incubated at 30 °C under shaking conditions. Samples were collected at regular intervals to monitor bacterial growth, 4-nitrophenol degradation and nitrite release. The bacterial growth was monitored by taking aborbance at 600 nm using spectrophotometer. For nitrophenol degradation, samples were cetrifuged and the degradation was monitored by taking optical density of supernatant at 420 nm. The nitrite release was monitored by a colourimetric method as described previously [2].

### 2.3 Bacterial DNA isolation

Strain PNP was cultured at 30 °C on nutrient agar plates. Under shaking conditions (180 rpm), a single colony of strain PNP was cultivated overnight in Nutrient broth. The pellet from the centrifuged bacterial culture was used to harvest DNA. The DNAminikit (Qiagen, Germantown, MD, USA) was used to extract genomic DNA according to the manufacturer's instructions.

**Table 2:** ANI and dDDH values of *Pseudomonas alloputida* strain PNP with its closely related type species exhibiting more than 98.7% 16S rRNA gene similarity.

S.No	Closely related Species	16S rRNA sequence similarity (%)	OrthoANIu value (%)	dDDH value (%)
1.	*Pseudomonas juntendi* BML3	99.79	87.57	34.70
2.	*Pseudomonas inefficax* JV551A3	99.79	89.35	38.9
3.	*Pseudomonas monteilii* NBRC 103158 = DSM 14164	99.73	89.40	39.0
4.	*Pseudomonas plecoglossicida* NBRC 103162 = DSM 15088	99.73	85.91	31.1
5.	*Pseudomonas asiatica* RYU5	99.73	89.22	39.0
6.	*Pseudomonas taiwanensis* DSM 21245	99.72	84.50	29.1
7.	*Pseudomonas entomophila* L48	99.66	84.55	29.2
8.	*Pseudomonas alloputida* Kh7	99.45	97.34	77.90
9.	*Pseudomonas mosselii* DSM 17497	99.38	84.86	29.5
10.	*Pseudomonas capeferrum* WCS358	99.31	86.37	31.80
11.	*Pseudomonas parafulva* NBRC 16636 = DSM 17004	99.25	84.79	29.0
12.	*Pseudomonas fulva* NBRC 16637	99.18	82.97	26.70
13.	*Pseudomonas sichuanensis* WCHPs060039	99.18	85.05	29.5
14.	*Pseudomonas guariconensis* strain LMG 27394	99.11	83.61	27.3
15.	*Pseudomonas putida* NBRC 14164	99.04	89.84	40.6
16.	*Pseudomonas reidholzensis* strain CCOS 865	98.97	83.95	28.10
17.	*Pseudomonas soli* strain LMG 27941	98.87	84.44	28.50
18.	*Pseudomonas aestus* CMAA1215	98.83	78.51	22.90
19.	*Pseudomonas piscis* MC042	98.83	78.53	22.90
20.	*Pseudomonas defluvii* WCHP16	98.83	79.46	23.60
21.	*Pseudomonas japonica* NBRC 103040	98.70	80.98	24.40

### 2.4 Whole genome sequencing, assembly and annotation

Following the manufacturer's instructions, a whole-genome sequencing library was created using the Nextera XT DNA library preparation kit.The HiSeq platform (Illumina, San Diego, CA, USA was used to sequence the libraries with 150 bp paired-end reads. The initial quality of the raw sequencing data was checked using FastQC [3] Trim galore 0.6.5 was used to trim the raw reads and adaptor con-tam-inations [4], and the Unicycler v0.4.8 was used for initial assembly [5]. Unless otherwise indicated, default parameters were used for all software. The NCBI Prokaryotic Genome Automatic Annotation Pipeline (PGAAP) [6] and the RAST server [7] were used for annotation. CGView server was used to create and visualise a graphical circular map of the entire genome [8].

### 2.5 16S rRNA gene sequence and phylogenetic analysis

RNAmmer software was used to extract the 16S rRNA gene sequence of strain PNP from its genome [9]. EzBiocloud evaluated the 16S rRNA gene sequence of strain PNP to determine its more closely related type strains [10]. The EzBiocloud database was used to obtain the 16S rRNA gene sequences of all closely related species. ClustalW was used to align all of the sequences [11]. The MEGA X software package was used to create a phylogenetic tree using the neighbour joining method [12].

### 2.6 Average nucleotide identity and digital DNA-DNA hybridization

The OrthoANI algorithm was used to calculate average nucleotide identity (ANI) amongs genomes of strain PNP [13] and its closely related species, and digital DNA-DNA hybridization (dDDH) values were calculated using genome-to-genome distance calculator (GGDC) 2.1.[14]

## Ethics Statement

No permission is required to carry out experiments for this manuscript.

## Data Availability

This Whole Genome Shotgun project has been submitted at DDBJ/ENA/GenBank under the accession JAGKJH000000000.

## CRediT authorship contribution statement

**Pankaj Kumar Arora:** Conceptualization, Investigation, Supervision, Writing – review & editing. **Raj Shekhar Saroj:** Conceptualization, Data curation, Formal analysis, Methodology, Resources, Software, Writing – original draft. **Rupali Mishra:** Conceptualization, Data curation, Formal analysis, Methodology, Resources, Software, Writing – original draft. **Rishabh Anand Omar:** Formal analysis, Methodology. **Puja Kumari:** Formal analysis. **Alok Srivastava:** Conceptualization, Investigation, Supervision, Writing – review & editing. **Sanjay Kumar Garg:** Supervision, Writing – review & editing. **Vijay Pal Singh:** Supervision, Writing – review & editing.

## Declaration of Competing Interest

The authors declare that they have no known competing financial interests or personal relationships that could have appeared to influence the work reported in this paper.
